# A bovine in vitro platform improves species comparability of mitochondrial apoptosis drug-response readouts

**DOI:** 10.1007/s00204-026-04506-9

**Published:** 2026-07-14

**Authors:** Frederike Werry, Axel Schöniger, Annett Honak, Janett Fischer, Paula Richter, Frank Edlich

**Affiliations:** 1 Faculty of Veterinary Medicine, Institute of Biochemistry, University of Leipzig, 04103 Leipzig, Germany; 2 Section of Hepatology, Department of Gastroenterology and Rheumatology, University Hospital Leipzig, Leipzig, Germany

**Keywords:** Intrinsic apoptosis, BAX, BAK, Outer mitochondrial membrane (OMM), Retrotranslocation

## Abstract

Species differences in mitochondrial apoptosis control can bias cytotoxicity endpoints and hazard-relevant mechanistic inference in preclinical test systems that rely on intrinsic-apoptosis readouts. We benchmarked steady-state localization and outer mitochondrial membrane (OMM) assemblies of the BCL-2 effectors BAX and BAK across mammals and combined this with functional apoptosis assays in bovine hepatocytes. Across species, bovine cells most closely recapitulated the human baseline state, whereas murine systems displayed distinct BAX/BAK partitioning and reduced capture of mitochondrial BAX in high-molecular-weight OMM assemblies. Functionally, bovine BFH12 cells showed minimal apoptotic response to the BH3 mimetic ABT-737 as a single agent, while responding robustly to doxorubicin and actinomycin D, as assessed by cytochrome* c* release, caspase processing and activity, and Annexin V/PI cytometry. These findings identify species-dependent regulation of BAX/BAK retrotranslocation and OMM assembly states as a baseline variable that shapes intrinsic-apoptosis pharmacodynamics. Incorporating bovine systems as a complementary non-rodent in vitro platform may improve model selection and pharmacodynamic readouts for compounds expected to engage mitochondrial apoptosis, thereby reducing avoidable translational misclassification in apoptosis-focused pipelines.

**Supplementary Information:**

The online version contains supplementary material available at https://doi.org/10.1007/s00204-026-04506-9.

## Introduction

Species differences in mitochondrial apoptosis control can systematically bias the interpretation of cytotoxicity and mechanism-of-action signals in preclinical test systems. This is particularly relevant for compounds that engage the intrinsic pathway, where the apparent magnitude and kinetics of cell death depend on how BCL-2 effectors are regulated at the outer mitochondrial membrane (OMM). If the underlying OMM signal transduction differs between commonly used model systems and humans, hazard inference, pharmacodynamic readouts, and model selection become vulnerable to false negatives or misleading translational expectations.

Here, we compared the regulation of the BCL-2 effectors BAX and BAK across mammalian species. BAX/BAK are controlled by retrotranslocation from the OMM into the cytosol, a process that differs markedly between humans and mice (Edlich et al. 2011; Schellenberg et al. 2013). We show that bovine cells recapitulate human-like OMM control of BAX/BAK localization, assembly into high-molecular-weight complexes, and pharmacologic responses to BH3 mimetics and cytotoxic agents, whereas murine systems diverge and can misreport drug effect. By anchoring compound responses in OMM signaling, our data define a mechanistic criterion for selecting preclinical systems and pharmacodynamic markers when interrogating mitochondrial/apoptotic liability, thereby improving the interpretability of in vitro and animal results in contexts where intrinsic apoptosis is a critical endpoint.

## Materials and methods

PBMCs from HCC patients and healthy donors, together with tissues from cattle, sheep, pig, and mouse, were used for cross-species benchmarking of BAX/BAK localization and OMM residency by selective permeabilization, fractionation, and immunoblotting; high-molecular-weight OMM assemblies were resolved by BN-PAGE. BFH12 cells served for functional bovine apoptosis assays, whereas MEF (WT/BAX-BAK DKO), U2OS DKO, and BV-173 cells were used for the respective mechanistic follow-up analyses, including DEVDase assays, cytochrome c release, PARP/caspase-3 cleavage, Annexin V/PI, live-cell SIM, and RT-qPCR. Detailed experimental methods are provided in SI Appendix.

## Results

**Fig. 1:**
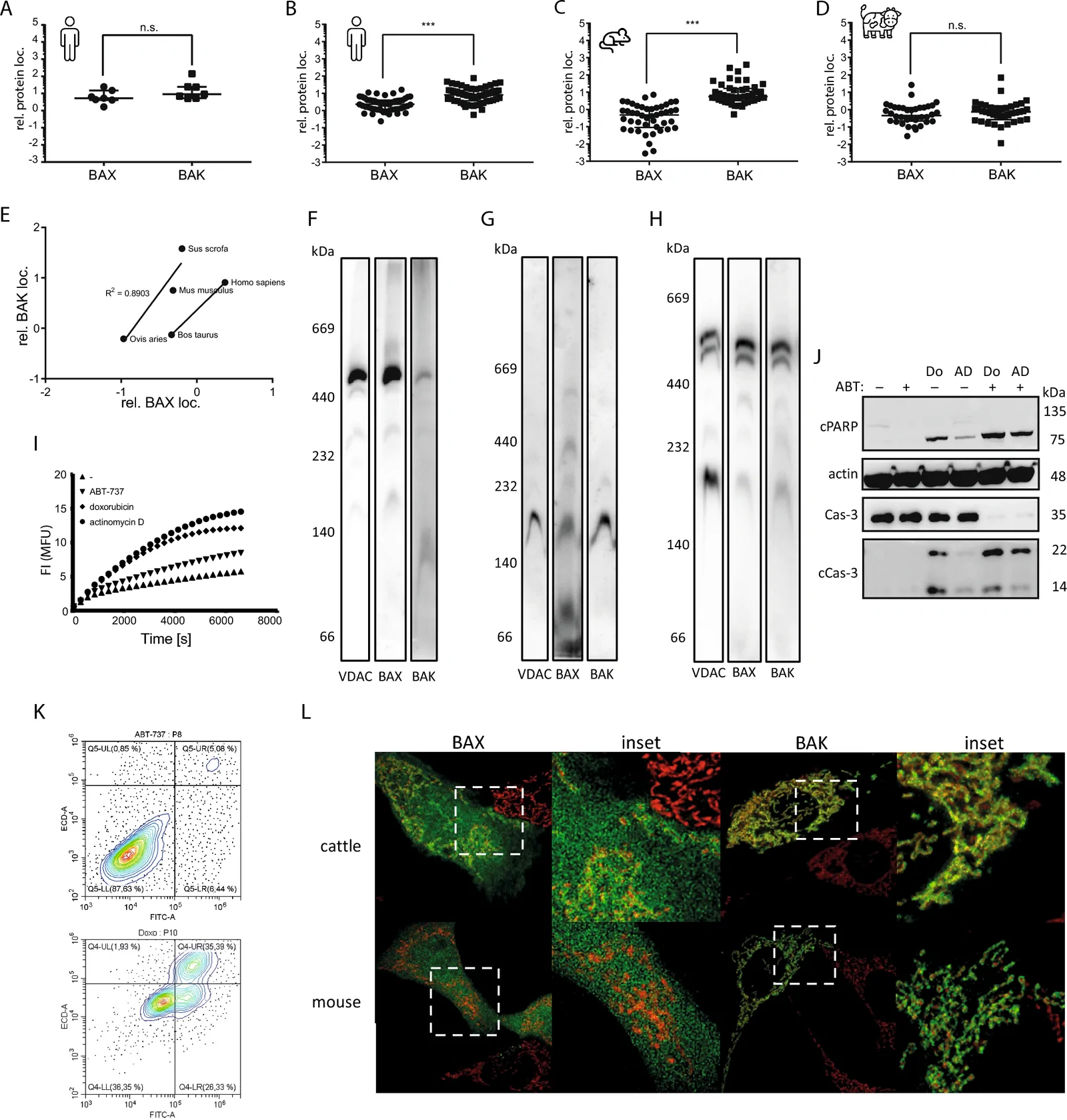
Species-specific BCL-2 control. **a**–**d** subcellular localization of BAX and BAK in PBMCs from healthy donors (a, n = 7), HCC patients (b, BAX n = 52; BAK n = 58), Mus musculus (c, n = 20), and Bos taurus (d, n = 3), quantified as log10(mitochondria-to-cytosol ratio). Two-tailed Mann–Whitney U tests as indicated. **e** species medians of relative BAX vs BAK (log10); median ± SD. **f–h** blue-native PAGE (BN-PAGE) of isolated mitochondria under basal conditions from human BV-173 BCR-ABL⁺ cells (f, n = 5), mouse MEFs/3T3 cells (g, WT; n = 5), and bovine skeletal muscle (h, n = 3). Immunodetection of VDAC, BAX, and BAK; apparent molecular masses indicated. **i–k** BFH12 2-2D bovine hepatocytes treated with ABT-737 (10 µM), doxorubicin (5 µM), or actinomycin D (1 µM): caspase-3 activity kinetics measured using Ac-DEVD-AFC (i, n = 4; technical duplicates; mean ± SD); immunoblots for PARP and caspase-3 cleavage and treatments combining ABT-737 with either doxorubicin (Do) or actinomycin D (AD) (j, n = 3; ACTIN loading control); Annexin V–FITC/propidium iodide flow cytometry after ABT-737 or doxorubicin (k, n = 3; quadrant percentages, mean ± SD). **l** structured-illumination microscopy (SIM) of live BFH12 2-2D and MEF BAX/BAK dKO cells expressing eGFP-BAX or eGFP-BAK under basal conditions; colocalization in yellow; SIM reconstruction. Inset boxes show magnified regions. Scale bars as indicated. n denotes biological replicates

To establish a molecular benchmark for species comparability, we quantified steady-state BAX and BAK localization across mammals using human PBMCs as the reference. This approach follows our previous work in AML and HCC, in which relative BAX/BAK localization consistently captured human apoptosis regulation and, in HCC, distinguished tumor from non-tumor tissue by the presence versus absence of a significant BAX-BAK separation (Reichenbach et al. 2017; Funk et al. 2020). BAX/BAK distribution was measured by selective permeabilization, subcellular fractionation, and quantitative immunoblotting and expressed as a log10 mitochondria-to-cytosol ratio. In the present dataset, PBMCs from HCC patients closely resembled healthy donor PBMCs, while again showing a significant BAX-BAK difference in the patient-derived context but not in healthy controls (Fig. 1a, b). Across all species, bovine cells most closely recapitulated the human pattern of BCL-2 effector control (Figs. 1c, d, S1a, b). At the amino acid sequence level, bovine BAX is markedly closer to human BAX than murine BAX, differing at only 2.6 percent of amino acid positions compared with 7.8 percent for the murine orthologue. In contrast to BAX, BAK appears overall less conserved across the species. In the human reference, BAK retained a cytosolic fraction under non-apoptotic conditions, whereas in mice BAK was shifted toward the OMM, a feature that was likewise observed in established murine cell lines, indicating that this murine baseline state was not confined to a single primary background. Across specimens, BAX/BAK localization spanned up to five orders of magnitude between cell populations. Notably, only bovine cells displayed a BAX/BAK ratio comparable to the human reference (Fig. 1e). Together, these differences define a species-dependent baseline for interpreting mitochondrial/apoptotic endpoints in assays that rely on BAX/BAK engagement.

To probe the underlying architecture, we analyzed OMM assemblies by blue native PAGE (BN-PAGE). In non-apoptotic human cells, BAX and BAK partition into ~ 500-kDa OMM assemblies previously linked to retrotranslocation control (Lauterwasser et al. 2016). Direct BN-PAGE analysis of human PBMCs, however, did not yield interpretable basal OMM assembly states, because the procedure itself imposed marked stress on these primary cells. We therefore turned to human AML cell lines as the most suitable available human system for this assay, combining technical robustness with a hematopoietic context closest to the PBMC-based analyses and continuity with our previous work on human apoptosis regulation (Reichenbach et al. 2017; Funk et al. 2020; Lauterwasser et al. 2016). In AML cells, BAX and BAK were recovered in the expected ~ 500-kDa range (Figs. 1f, S1c). Interspecies organization was conserved in principle but differed in size distribution and the fraction of mitochondrial BAX/BAK captured in high-molecular-weight assemblies. In sheep, both BAX and BAK were detected in smaller assemblies (Fig. S1d), whereas in mice mitochondrial BAX was predominantly found outside the ~ 500-kDa range, enriched in lower-molecular-weight species (Fig. 1g). These complex sizes were observed in both murine backgrounds examined here, C57BL/6J and 129, indicating that the murine assembly pattern was not restricted to a single genetic background, consistent with our findings in additional murine cell models (Fig. S1e). By contrast, bovine cells closely matched the human pattern, with the majority of mitochondrial BAX and BAK recovered within high-molecular-weight assemblies (Figs. 1h, S1f). Elevated anti-apoptotic BCL-2 coincided with retained mitochondrial BAX/BAK localization (Fig. S1g), consistent with a high basal retrotranslocation rate rather than loss of OMM engagement (Fig. S1h–j). Collectively, these data indicate that species-dependent OMM assembly states of BAX/BAK constitute a distinct baseline variable that is likely to influence how intrinsic-apoptosis endpoints report cellular stress and compound exposure.

Functionally, we assessed intrinsic-apoptosis readouts in bovine fetal hepatocyte-derived BFH12 cells (Fig. S1k). Caspase-3 activity (Ac-DEVD-AFC) increased rapidly after doxorubicin and actinomycin D, but showed only marginal and delayed induction after the BH3 mimetic ABT-737 (Fig. 1i). In line with this profile, ABT-737 did not produce detectable cytosolic cytochrome *c* accumulation, whereas doxorubicin and actinomycin D induced robust release (Fig. S1l). To capture endpoint processing, immunoblotting for cleaved caspase-3 and cleaved PARP likewise showed minimal ABT-737-associated cleavage in BFH12, whereas combining ABT-737 with either doxorubicin or actinomycin D enhanced cleavage beyond the respective single-agent treatments (Figs. 1j, S1m). Annexin V/PI flow cytometry corroborated these results: approximately 85% of ABT-737–treated cells remained Annexin V⁻/PI⁻, while doxorubicin caused rapid, extensive apoptosis and actinomycin D a milder but reproducible increase in Annexin V positivity (Figs. 1k, S1n). Finally, structured-illumination microscopy in living BFH12 cells revealed a discrete mitochondrial BAX pool under non-apoptotic conditions, whereas BAK localization appeared comparable between bovine and murine cells (Fig. 1l). Together with the species-benchmark and BN-PAGE data, these functional readouts support bovine cells as a human-like system for OMM-centered BAX regulation and intrinsic-apoptosis pharmacodynamics.

## Conclusion

In summary, our data identify species-dependent baseline differences in OMM-centered BAX/BAK control as a concrete source of bias in preclinical systems that interrogate intrinsic-apoptosis endpoints. Across benchmarks of steady-state localization and BN-PAGE assemblies, bovine cells most closely matched the human reference pattern, whereas murine systems displayed distinct baseline states. This observation does not diminish the central value of rodent models for many biological questions, but it underscores that interspecies differences in baseline apoptosis regulation—together with broader differences in stress-response determinants such as DNA repair and microenvironmental context—can constrain direct extrapolation when the intended endpoint is mitochondrial apoptosis (Xu et al. 2022; Riva et al. 2020; Holliday 1996; Bailey et al. 2014). Functionally, bovine BFH12 cells showed limited responsiveness to the BH3 mimetic ABT-737 as a single agent, but clear enhancement of apoptosis induction when ABT-737 was combined with cytotoxic agents, consistent with a regulatory constraint upstream of effector activation rather than general apoptosis incompetence. Accordingly, explanations that rely solely on cross-compensation among anti-apoptotic BCL-2 proteins appear incomplete, since functional redundancy is also present in settings in which venetoclax (ABT-199) can be effective. Our results instead highlight OMM-level control of BAX/BAK, including retrotranslocation and assembly states, as a tractable mechanistic variable that can shape pharmacodynamic readouts. We therefore propose that species-divergent regulation of BAX/BAK retrotranslocation at the OMM should be treated as an explicit model-selection variable and as a mechanistic anchor for pharmacodynamic endpoints when evaluating compounds expected to engage mitochondrial apoptosis. Incorporating bovine systems as a complementary non-rodent in vitro platform may improve interpretability and reduce avoidable translational misclassification in apoptosis-focused preclinical pipelines.

## Supplementary Information

Supplementary Material 1.

## Data Availability

The data generated in this study are available within this article and its Supplementary Materials.
