# Supplementary material for: A bovine in vitro platform improves species comparability of mitochondrial apoptosis drug-response readouts
Source: Arch Toxicol. 2026 Jul 14;100(11):5159–63. doi: 10.1007/s00204-026-04506-9 (PMC13694350; doi:10.1007/s00204-026-04506-9)
Supplement: Supplementary file 1 [file 204_2026_4506_MOESM1_ESM.pdf]

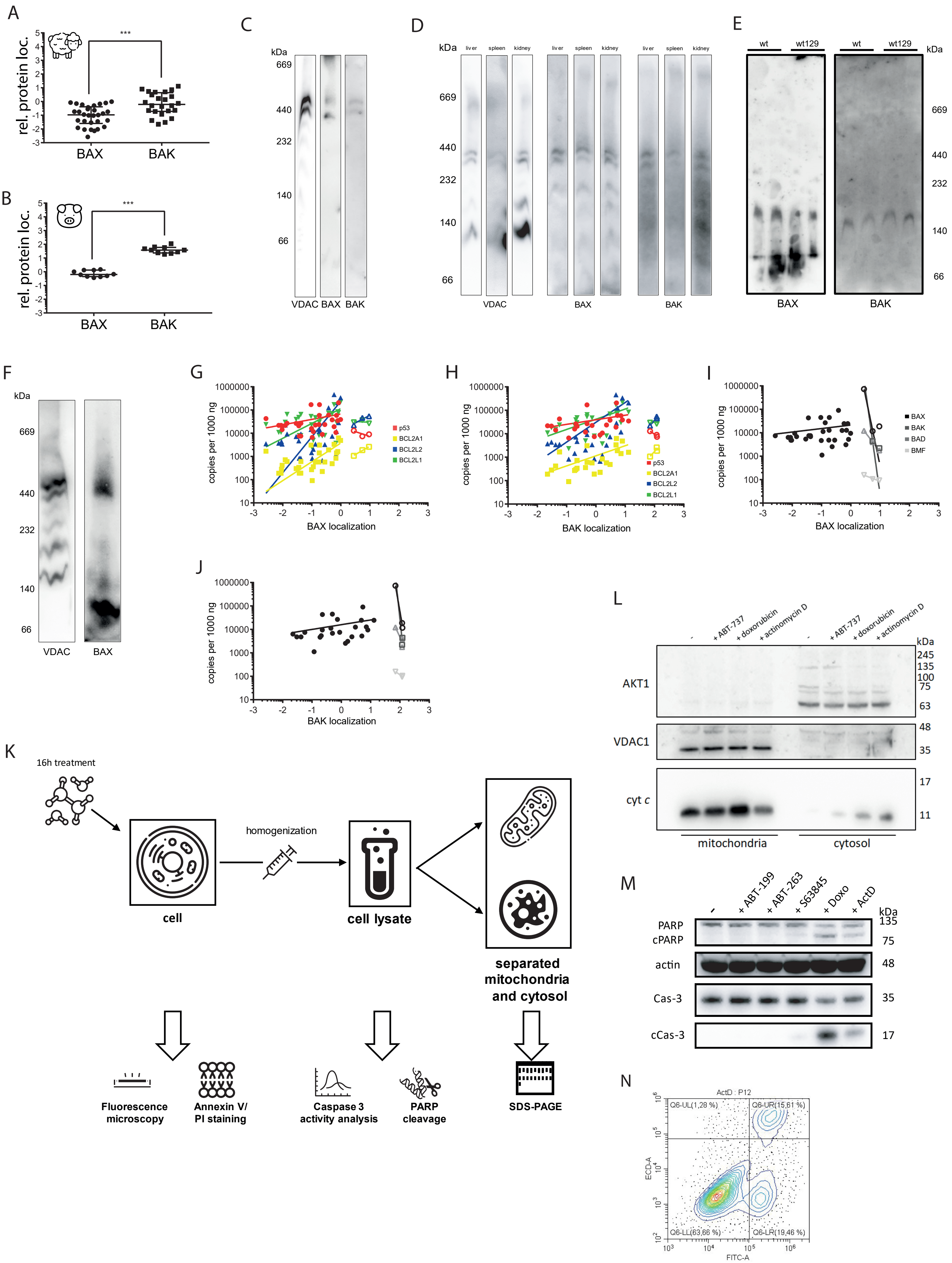
A
rel. protein loc.
***
BAX
BAK
B
rel. protein loc.
***
BAX
BAK
C
kDa
669
440
232
140
66
VDAC
BAX
BAK
D
kDa
669
440
232
140
66
liver
spleen
kidney
VDAC
BAX
BAK
E
wt
wt129
kDa
669
440
232
140
66
BAX
BAK
F
kDa
669
440
232
140
66
VDAC
BAX
G
copies per 1000 ng
BAX localization
p53
BCL2A1
BCL2L2
BCL2L1
H
copies per 1000 ng
BAK localization
p53
BCL2A1
BCL2L2
BCL2L1
I
copies per 1000 ng
BAX localization
BAX
BAK
BAD
BMF
J
copies per 1000 ng
BAK localization
K
16h treatment
cell
homogenization
cell lysate
separated mitochondria and cytosol
Fluorescence microscopy
Annexin V/ PI staining
Caspase 3 activity analysis
PARP cleavage
SDS-PAGE
L
-
+ ABT-737
+ doxorubicin
+ actinomycin D
kDa
AKT1
VDAC1
cyt c
245
135
100
75
63
48
35
17
11
mitochondria
cytosol
M
-
+ ABT-199
+ ABT-263
+ S63845
+ Doxo
+ ActD
kDa
PARP
cPARP
actin
Cas-3
cCas-3
135
75
48
35
17
N
ActD : P12
Q6-UL(1,28 %)
Q6-UR(15,61 %)
Q6-LL(63,66 %)
Q6-LR(19,46 %)
ECD-A
FITC-A

Supplementary Figure 1 | Extended analyses supporting species-dependent BCL-2 regulation. **(a, b)** Relative subcellular localization (log10[mitochondria:cytosol]) of BAX and BAK in *Ovis aries* (a; n=8) and *Sus scrofa* (b; n=10); two-tailed Mann–Whitney U tests.
(c-f) Blue-native PAGE (BN-PAGE) of isolated mitochondria under basal conditions from human 697 cells (c, n=3), sheep liver, spleen, and kidney (d, n=3), C57BL/6J (wt) and 129 (wt129) MEFs (e, n=3), and bovine liver (f, n=3). Immunodetection of VDAC, BAX, and BAK; apparent molecular masses indicated.
(g–j) *Ovis aries* (solid) *Bos taurus* (open)*:* scatter plots of relative BAX or BAK localization (log10) versus qPCR transcript copies per 1,000 ng RNA for the indicated genes (TP53, BCL2A1, BCL2L2, BCL2L1; color-coded as in the plots) and, where indicated, pro-apoptotic transcripts (BAX, BAK, BAD, BMF).
(k) Schematic linking BFH12 2-2D bovine hepatocyte assays to main-figure panels (Fig. 1i–k) and to Fig. S1l-n.
(l) Western blots of cytochrome c release after apoptosis induction (ABT-737 10 µM; doxorubicin 5 µM; actinomycin D 1 µM); cytosolic and heavy-membrane fractions validated by AKT1 (cytosol) and VDAC1/Porin (mitochondria); apparent molecular mass (kDa) indicated; n=3.
(m) BFH12 bovine hepatocytes treated with ABT-199 (1 µM), ABT-263 (1 µM), S63845 (1 µM), doxorubicin (5 µM), or actinomycin D (1 µM): immunoblots for PARP and caspase-3 cleavage from the same samples (n=3; ACTIN loading control).
(n) Annexin V–FITC/propidium iodide (PI) flow cytometry after actinomycin D (1 µM); quadrant percentages shown; n=3.
n denotes biological replicates.

Methods

Cell culture and transfection

U2OS BAX/BAK double-knockout (DKO) cells were maintained in McCoy's 5A (Gibco) supplemented with 10% (v/v) fetal bovine serum (FBS; Biochrom) and 10 mM HEPES (pH 7.4; Roth). BV-173 cells were cultured in RPMI-1640 (Gibco) with 10% FBS (Biochrom). MEF wild-type and MEF BAX/BAK DKO cells were grown in high-glucose DMEM (Gibco) with 10% sterile-filtered FBS (Biochrom) and 4 mM L-glutamine (Biochrom). BFH12 clone 2-2D cells were cultured in William's E medium (Biochrom) with 10% heat-inactivated, sterile-filtered fetal calf serum (FCS; Biochrom), 2 mM L-alanyl-L-glutamine (Biochrom), 100 nM dexamethasone, 4,27 mg insulin (SIGMA-Aldrich), and 1 g glucose. All cells were kept at 37 °C in 5% CO_2. Transfections were performed with TurboFect (Thermo Scientific) or Lipofectamine LTX (Invitrogen) using pEGFP-BAX or pEGFP-BAK constructs according to the manufacturers' instructions[1].

Sample preparation

Human peripheral blood mononuclear cells (PBMCs) were obtained from patients with hepatocellular carcinoma (HCC) at the Research Laboratory for Clinical and Experimental Hepatology, University Hospital Leipzig, before and after treatment (systemic: lenvatinib, sorafenib, or regorafenib; locoregional: TARE or TACE). Animal tissues (*Ovis aries, Bos taurus, Sus scrofa*) were sourced from slaughtered animals via institutional collaborations; surplus samples from *Mus musculus* were used. Samples were stored at −80 °C until use.

Western blotting

Proteins were resolved by SDS–PAGE and transferred to nitrocellulose (0.2 µm; Amersham) at 400 mA for 90 min (wet transfer). Membranes were blocked and probed overnight (4 °C) with primary antibodies: anti-BAX (Proteintech, 50599-1-AP), anti-BAK (SIGMA B5897), anti-AKT1 (Proteintech, 60203-2-Ig), anti-VDAC1 (Proteintech, 55259-1-AP), anti-cytochrome c (Proteintech, 10993-1-AP), anti-PARP (Cell Signaling, 9542S), anti-ACTIN (Merck, MAB1501), anti-caspase-3 (Cell Signaling, 9662S), and anti-cleaved caspase-3 (Cell Signaling, 9664S), followed by HRP-conjugated secondary antibodies. Blots were washed in PBS-T (0.05% Tween-20) and imaged on a Fusion X system (Vilber) using FUSION Capt Edge software. Fraction purity was controlled with AKT1 (cytosolic) and VDAC1 (mitochondrial) markers. Densitometry was performed with the integrated quantification tool.

Caspase-3 activity assay

BFH12 2-2D cells were seeded in 15-cm dishes and treated for 16 h with 10 µM ABT-737 (Hycultec), 5 µM doxorubicin (Sigma-Aldrich), or 1 µM actinomycin D (Sigma-Aldrich). Cells were scraped in ice-cold PBS, homogenized twice through a 25G needle (Braun), and cleared (2,000 × g, 5 min, 4 °C). Lysates (100 µL) were transferred to OptiPlate-96F (PerkinElmer). Protein concentrations (Bradford; Roth) were used for normalization. Reactions contained 50 µM Ac-DEVD-AFC (BD Pharmingen) and 2 mM DTT (Roth). Substrate cleavage kinetics were recorded in duplicate for 150 cycles at 37 °C (Ex 380/20 nm, Em 460/30 nm) and fitted to activity curves (SigmaPlot).

Flow cytometry (Annexin V/PI)

Supernatants were collected; adherent cells were rinsed with PBS and detached by trypsinization (8 min). Supernatants and cells were combined and pelleted (1,000 × g, 5 min). After PBS wash, 3×10^5 cells were

stained with the eBioscience™ Annexin V Apoptosis Detection Kit FITC (Thermo Fisher Scientific) per manufacturer's instructions. Briefly, cells were washed in binding buffer, incubated with Annexin V-FITC (20 min, RT, dark), washed, resuspended in binding buffer, and stained with PI. Acquisition was performed within 1 h on a CytoFLEX S (Beckman Coulter) using CytExpert software. Instrument QC was performed before each run with CytoFLEX Daily QC Fluorospheres (Beckman Coulter). Annexin V-FITC was detected with a 488 nm laser/525/40 nm filter; PI with a 561 nm laser/610/20 nm filter. A minimum of 2×10^5 events were recorded per sample. Debris and doublets were excluded (SSC-A/FSC-A; FSC-H/FSC-A). Quadrant gating (FITC-A vs. ECD-A) defined viable (ANX V^-/PI^-), early apoptotic (ANX V^+/PI^-), late apoptotic (ANX V^+/PI^+), and necrotic (ANX V^-/PI^+) populations.

Whole-Cell lysis and subcellular fractionation

Cell pellets were resuspended in SEM buffer (10 mM HEPES, 250 mM sucrose, pH 7.2) with protease inhibitors (5 µg/ml Cytochalasin B, Enzo; 10 µg/ml Aprotinin, Roth; 50 µg/ml Leupeptin, Enzo; 400 ng/ml Pepstatin A, Sigma-Aldrich; 100 µM PMSF, Sigma-Aldrich; 8 µM MG132, Enzo; 300 µM AEBSF, Roche) and incubated 10 min on ice. Homogenization was performed with a 25G needle, followed by clarification (2,000 × g, 2 × 5 min, 4 °C). Supernatants were centrifuged (15,000 × g, 60 min, 4 °C) to separate heavy membranes (mitochondria) from cytosol.

Tissue samples were erythrocyte-lysed (RBC lysis buffer, eBioscience; 3 × 10 min). Soft tissues (liver, spleen, kidney) were Dounce-homogenized in SEM; muscle was processed with a disperser (IKA T-25 Ultra-Turrax). Heavy membranes were pelleted (15,000 × g, 60 min, 4 °C).

Mitochondrial pellets were washed (SEM, 3×). Supernatants were further clarified (20,800 × g, 30 min, 4 °C) to obtain purified cytosol. Samples were mixed with SDS sample buffer (final: 7 mg/mL DTT, 10 mM Tris-HCl, 1 mM EDTA, 13% glycerol, 3% SDS, 0.01% bromophenol blue), resolved by SDS–PAGE (4–10%), and analyzed by Western blotting.

Native complex analysis (BN-PAGE)

Mitochondria-enriched heavy membranes were solubilized in 1% digitonin buffer (20 mM Tris-HCl pH 7.4, 50 mM NaCl, 0.1 mM EDTA, 10% glycerol, 1% [w/v] digitonin, 1 mM PMSF) for 30 min at 4 °C; insoluble material was removed (15,000 × g, 5 min, 4 °C). Extracts were supplemented with 10× Blue Native loading dye (500 mM EACA, 100 mM Bis TRIS, 5 % Coomassie brilliant blue G250) and separated on 4–13% gradient gels. Complexes were transferred to PVDF membranes for semidry immunoblotting.

Real-time PCR

Total RNA was isolated from 25 mg bovine or ovine tissue using the RNeasy Lipid Tissue Mini Kit (Qiagen, 74804) per manufacturer's protocol, following homogenization (IKA T-25 Ultra-Turrax) and column shredding. DNase treatment was performed with the TURBO DNA-free™ Kit (Ambion, AM1907). RNA quantity/quality was assessed on an NP80 spectrophotometer (Implen); samples were stored at −80 °C. cDNA was synthesized from 1,000 ng RNA using ProtoScript II First Strand cDNA Synthesis Kit (NEB, E6560). qPCR was performed with SensiMix SYBR No-ROX Kit (Meridian Bioscience, QT650-05) on a Rotor-Gene Q (Qiagen) using SYBR® Green detection. Cycling: 95 °C 10 min; 45 cycles of 95 °C 15 s, 58 °C 25 s (annealing), 72 °C 15 s; melting curve 50–99 °C. Reference genes included ACTB, GAPDH, and (verify: 18S or 28S rRNA).

Structured illumination microscopy (SIM)

Lattice SIM was performed on an Elyra 7 system (Zeiss) with an Axio Observer 7 SR stand and a Plan-Apochromat 63×/1.4 oil objective. Live-cell imaging was done at 37 °C, 5% CO_2 with humidity control. Acquisition in Lattice SIM mode captured 15 phase images per exposure (488 nm for eGFP; 642 nm for MitoTracker Deep Red FM, Invitrogen) with two camera channels (TV1: BP 570–620 + LP 655; TV2: BP 420–480 + BP 495–550). Images were acquired in 16-bit with 4-frame averaging, reconstructed with ZEN (SIM2 algorithm), and projected by maximum intensity. Colocalization was quantified by Pearson's correlation coefficient.

Cell lines and sources

BFH12 cells are an SV40 large T-antigen-transduced extended-passage bovine fetal hepatocyte-derived cell line, originally established and characterized by Gleich et al[2]. Cells were used at passages 6–20 in the present study. U2OS BAX/BAK DKO cells were acquired from Stefan Jakobs (MPI-BPC, Göttingen). MEF cells were acquired from Richard Youle (NIH, Bethesda). Authentication and routine mycoplasma testing were performed for all cell lines used in this study.

References

1. Lauterwasser, J. *et al.* The porin VDAC2 is the mitochondrial platform for Bax retrotranslocation. *Sci. Rep.* **6**, 32994 (2016).
2. Gleich, A., Kaiser, B., Schumann, J. & Fuhrmann, H. Establishment and characterisation of a novel bovine SV40 large T-antigen-transduced foetal hepatocyte-derived cell line. *Vitro Cell. Dev. Biol. - Anim.* **52**, 662–672 (2016).
